# Human Gut Microbes Associated with Systolic Blood Pressure

**DOI:** 10.1155/2022/2923941

**Published:** 2022-02-03

**Authors:** Tulsi Kumari Joishy, Aashish Jha, Mai Oudah, Santanu Das, Atanu Adak, Dibyayan Deb, Mojibur Rohman Khan

**Affiliations:** ^1^Molecular Biology and Microbial Biotechnology Laboratory, Life Sciences Division, Institute of Advanced Study in Science and Technology (IASST), Guwahati, Assam, India; ^2^Department of Molecular Biology and Biotechnology, Cotton University, Guwahati, Assam, India; ^3^Genome Heritage Group, Program in Biology, New York University Abu Dhabi, Abu Dhabi, UAE; ^4^Program of Computer Science, New York University Abu Dhabi, Abu Dhabi, UAE

## Abstract

Emerging studies have revealed a strong link between the gut microbiome and several human diseases. Since human gut microbiome mirrors variations in lifestyle and environment, whether associations between disease conditions and gut microbiome are consistent across populations—particularly in communities practicing traditional subsistence strategies whose microbiomes differ markedly from industrialists—remains unknown. Cardiovascular diseases are the leading cause of mortality in India affecting 55 million people, and high blood pressure is one of the primary risk factors for cardiovascular diseases. We examined associations between gut microbiome and blood pressure along with 14 other variables associated with lifestyle, dietary habits, disease conditions, and clinical blood markers in the three Assamese populations. Our analysis reveals a robust link between the gut microbiome diversity and composition and systolic blood pressure. Moreover, several genera previously associated with hypertension in non-Indian populations were also associated with systolic blood pressure in this cohort and these genera were predictors of elevated blood pressure in these populations. These findings confer opportunities to design personalized, preventative, and targeted interventions harnessing the gut microbiome to tackle the burden of cardiovascular diseases in India.

## 1. Introduction

The human gut comprises diverse microbial community collectively known as the gut microbiome (GM), which plays a crucial role in human health and diseases [1]. GM is shaped by various factors, including diet, lifestyle, medication, environment, and genetics [2, 3], and influences human physiology, metabolism, and immune responses [4, 5]. Studies have shown a link between GM and etiology of several chronic diseases [1, 6, 7], and identifying the fecal microbial markers specific to diseases is an emerging and powerful tool for developing preventative approaches as well as early diagnosis and treatment [8, 9].

Hypertension is a major risk factor for cardiovascular, cerebrovascular, and kidney diseases [10, 11]. The etiology of hypertension depends on the complex interplay of genetic, environmental, and dietary factors [12, 13]. Diet strongly influences the GM, which makes it likely that alterations in GM and its functions might contribute to the development of hypertension. Indeed, several studies have shown associations between blood pressure and GM in humans [1, 8, 14–17]. Compared to individuals with normal blood pressure, the GM in hypertensive patients has decreased microbial diversity and increased Firmicutes-to-Bacteroidetes ratio. Some of the observations linking GM to hypertension have also been replicated in animal models [14–16, 18]. However, most of the studies investigating the role of GM on the etiology of hypertension and other chronic diseases have focused on industrialized populations such as Americans [1, 17], Europeans [1, 16], and Chinese living in modern cities [8]. Given the differences in the GM across human populations, whether these results are applicable to non-Western or traditional communities remains unexplored. For example, consumption of milk fermented with *Lactobacilli* has been reported to lower blood pressure in certain human populations [8, 15, 19]. However, despite frequent consumption of *dahi/doi* (yogurt), which contains high dosages of *Lactobacilli* [20], hypertension has historically remained a major problem in India. At present, 45% of the Indian adults, which amounts to a staggering 1.3 billion individuals, are estimated to have elevated blood pressure [21] and are at risk of developing cardiovascular diseases. Recent statistics suggest that 1 in 4 deaths in India are due to cardiovascular diseases [22]. Therefore, identifying gut microbial features associated with high blood pressure can lead to novel preventative and therapeutic approaches that are urgently needed to address the high burden of cardiovascular disease in India.

Here, we evaluated the relationship between the gut microbiome and 22 host factors, including blood pressure in 71 healthy Assamese individuals from three rural villages in Assam, a Northeastern Indian state with high prevalence of hypertension [23, 24]. Two of the study sites, Aanthmile and Jagiroad, were home to Nepali speakers who have historically resided in Assam for generations, exhibit an agropastoralist lifestyle typical of Assam, and are major dairy producers in Assam. The third location, Kamalabari (Majuli), was home to the Satra, a group of native Assamese males who practice animal herding and follow a spiritual lifestyle governed by the Neo-Vaishnavite code of conduct [25]. We found subtle differences in dietary and lifestyle practices in these geographically cohabiting populations, which explained a relatively small fraction of gut microbial diversity and composition between these populations. After accounting for lifestyle, we were able to identify a robust association between different measures of gut microbiome and systolic blood pressure in these individuals. Furthermore, using a machine learning approach, we were able to identify several bacterial taxa that are predictive of elevated blood pressure in this Indian population.

## 2. Materials and Methods

### 2.1. Study Sites, Participating Individuals, and Sample Collection

Fecal and blood samples were collected with written informed consent from a total of 71 individuals within Assam, India. Participants were from three geographic locations, namely, Aanthmile (*N* = 24), Jagiroad (*N* = 24), and Kamalabari, Majuli (*N* = 23). Fresh fecal samples were collected in sterile stool collection tubes containing RNAlater™ (Cat. No. 76104, Qiagen, Germany) solution to maintain the integrity of DNA. The tubes were shaken hard in a back and forth motions until the samples were homogenized. Blood samples were collected using K3 EDTA (tripotassium ethylenediaminetetraacetic acid) vials, and serum was separated immediately by centrifugation at 3000 rpm for 5 min at room temperature. Additionally, anthropometric measures including height, weight, body mass index, and blood pressure measures were also determined from each participant, while sample collection on-site participants also filled out a basic survey questionnaire that assessed their age, gender, diet (the primary source of carbohydrates, proteins, and vegetables), consumption of dairy products, alcohol, health status, and use of medication.

### 2.2. DNA Extractions and 16S rDNA Gene Amplicon Sequencing

Microbial DNA was extracted from 200 *μ*L homogenized fecal samples using Qiagen DNA Stool Mini-Kit (Qiagen, Hilden, Germany) according to the manufacturer's protocol. This extraction protocol involved chemical lysis followed by heating at 95°C for 15 mins for recovery of Gram-positive bacteria as well. Quantification of double-stranded DNA was determined using the QuantiFluor dsDNA System (Promega, Wisconsin, USA), and an estimate of sample purity was determined via spectrophotometry by measuring the A260/A280 absorbance ratio. The V3+V4 region of the 16S rDNA gene was PCR amplified and sequenced at Macrogen Inc. (Seoul, Republic of Korea) using the 341F-805R primer pairs following the standard Illumina protocols. The amplified DNA fragments were subjected to paired-end (2 × 300 bp) sequencing using the MiSeq platform (Illumina). Gut microbiome sequencing data were obtained in the form of FASTQ files.

### 2.3. 16S rRNA Data Analysis

Read counts for amplicon sequence variants (ASVs) were calculated using the phyloseq pipeline [26] and the Silva 138 database training set, [27]. FASTQ files were used to assess the quality of the sequence reads [28]. High-quality sequences were retained after trimming reads to 265 bases (removing 10 bp from the start of each read and trimming at the 275th base) from both the forward and reverse reads, respectively. Filtering and trimming were performed allowing maximum of 2 expected errors {maxN = 0, truncLen = c (290,280), maxEE = c(2,2), truncQ = 2}. Paired-end reads were merged together to create a table containing amplicon sequence variant counts. Taxonomy was assigned using a naïve Bayesian classifier method [29] implemented in the DADA2 algorithm [28]. Multiple alignment was performed using the DECIPHER package [30], and phangorn was used to construct maximum likelihood phylogenetic tree. Finally, a phyloseq object was constructed by merging tables containing ASV counts, taxonomic hierarchy, sample data, and phylogenetic tree. After removing singletons and lowly abundant (present in less than 5% of individuals) ASVs, a total of 1935 ASVs remained and were used for subsequent analysis.

### 2.4. Blood Biochemical Tests

Serum samples were analyzed using standard biochemical assay kits from Coral Clinical Systems (Salcete, Goa) following the manufacturer's protocol. The tests included 6 blood parameters, namely, albumin, globulin, total protein, glucose, cholesterol, and triglycerides levels.

### 2.5. Statistical Analysis

All statistical analyses were conducted using R version 3.5.1 [31]. The survey data and the blood parameters were collectively assessed using principal component analysis (PCA). Phylogenetic diversity was assessed using two measures of alpha diversity, namely, species richness and Shannon's H, computed by rarefying the samples to various depths starting from 2,500–30,000 sequences per sample. The maximum depth of 30 k allowed for the inclusion of all samples. Alpha diversity was compared using species richness and Shannon's H at the rarefaction depth of 30,000 using Kruskal–Wallis tests followed by Dunn's post hoc test to assess pairwise differences. Beta diversity was assessed using Bray–Curtis as well as unweighted and weighted UniFrac distances. PERMANOVA was performed using the vegan package [32], and 10,000 randomizations were performed to assess the statistical significance. The differences in taxa abundance (counts) were assessed using the DESeq2 package [33].

Three separate generalized linear mixed models were used to evaluate associations between the blood parameters, dietary factors, location, and different features of the gut microbiome, namely, species richness, Shannon's diversity index, and PCo1 (using Bray–Curtis distance). In all the three models, the gut microbiome features were treated as the response variable, variables linked to diet, lifestyle, and blood were treated as fixed effects, and samples were treated as random effects. A total of 15 factors were treated as explanatory variables with fixed effects, and they were as follows: location, age, sex, milk drinking pattern, gastric condition, medicinal use, systolic blood pressure, diastolic blood pressure, hypertension, BMI, albumin, globulin, triglycerides, cholesterol, and RBS glucose. All statistical tests with *P* < 0.05 after multiple testing corrections using the Benjamini–Hochberg false discovery rate adjustment were considered statistically significant.

### 2.6. Machine Learning Predictive Models

Individuals were binned into two groups based on their systolic blood pressure. Individuals with systolic blood pressure <120 mmHg and ≥ 120 mmHg were considered normal and elevated, respectively. All of the 1,935 ASVs were used, and the microbiome of each sample was represented via the feature vector of their relative abundances. As a preprocessing step, the hierarchical feature engineering (HFE) method [34] was used. HFE exploits the intrinsic hierarchical structure of the feature space to generate a small set of informative features that can be used for classification. Following the identification of the final informative feature set, we used Waikato Environment for Knowledge Analysis (WEKA) [35] for classification. Three ML approaches—random forest, naive Bayes, and decision trees algorithms—were used for classification by partitioning the individuals into a training (70%) and test (30%) sets and using 10-fold crossvalidation in order to avoid model overfitting.

## 3. Results

### 3.1. Lifestyle and Dietary Characteristics of Study Participants

**Figure 1:**
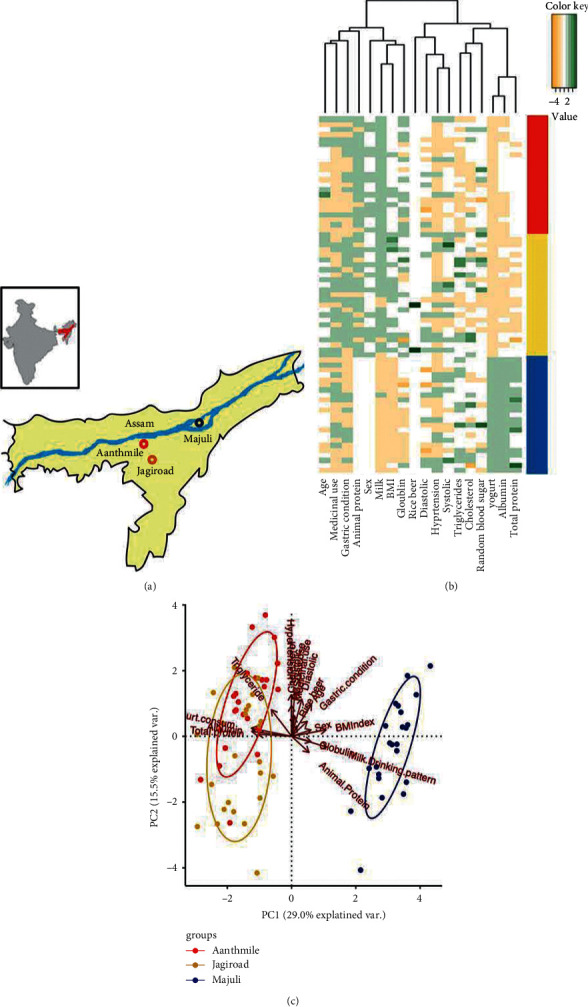
Lifestyle, dietary, and clinical blood markers differ between Assamese populations. (a) Map showing the three different sampling locations in Assam. Aanthmile (red) and Jagiroad (gold) are home to Nepali speakers who practice agropastoralism. Kamalabari, Majuli (blue), is home to the native Assamese known to practice traditional animal herding and meditation. (b) A heatmap showing differences in diet as well as blood phenotypes between these populations. (c) A principal component analysis (PCA) differentiates populations based on their diet and clinical blood markers. PC1 separates individuals by lifestyle/diet and body mass index (BMI). PC2 is associated with several risk factors for chronic diseases, including age, systolic blood pressure as well as disease conditions, e.g., diabetes and gastric condition (presence or absence of gastritis) that do not differ between the two lifestyles.

Located in the foothills of the Himalaya, Assam is home to various indigenous ethnic groups that continue to practice traditional subsistence strategies. We collected samples from 71 individuals from three rural villages on the banks of the Brahmaputra River, namely, Aanthmile, Jagiroad, and Kamalabari (Figure 1(a)). The residents of Aanthmile and Jagiroad are representative of Indians of Indo-European descent spread across India. They practice agropastoralism and are the major dairy producers in Assam. Kamalabari is home to Satra, a community of native Assamese animal herders that are known for their practice of Neo-Vaishnavite lifestyle.

We assessed the impact of lifestyle and dietary habits on these populations using a survey questionnaire that included 22 questions pertaining to current dietary practices such as primary carbohydrate and protein source, consumption of animal products (frequency of meat and dairy products), medicinal usage, and medical conditions such as presence or absence of gastritis. Participants included 20 females (28.2%) with average age of 35.4 ± 11.6 years and an average BMI of 24.5 ± 6.1 kg/m^2^. The 51 males (71.8%) were 35.9 ± 11.4 years old with an average BMI of 19.00 ± 7.9 kg/m^2^ (Table 1). Participants had an average systolic blood pressure of 122.32 ± 16.47 mmHg. Mean systolic blood pressure in females and males was 122.3 ± 13.26 mmHg and 122.3 ± 17.69 mmHg, respectively. Similarly, mean diastolic blood pressure in females and males was 73.6 ± 10.18 mmHg and 70.52 ± 12.44 mmHg, respectively. Analysis of the survey data revealed subtle variations in diet among the participants from the three sampling locations (Figures 1(b) and 1(c)). For all participants, rice was the major source of carbohydrates, while milk products and dal (legumes) were the primary sources of protein. All participants consumed fiber-rich greens, vegetables, and fruits daily. Consumption of meat was rare, but participants of Aanthmile and Jagiroad consumed meat occasionally and those of Kamalabari consumed fish but not meat. Most participants consumed *doi* (yogurt) on a daily basis, although the residents of Aanthmile and Jagiroad consumed *doi* made by propagating cultures of boiled cow milk, whereas the residents of Kamalabari consumed *doi* prepared by natural fermentation of raw cow milk. Few phenotypes and biochemical parameters, including BMI, blood albumin, globulin, and total protein, varied between locations. Albumin and total protein serve as markers of nutrition, while the higher concentration of globulin is indicative of higher pathogen load [36, 37]. Furthermore, BMI was positively correlated with age (Spearman's rho = 0.486, *P*=1.708*e* − 05) and it was significantly associated with clinical blood markers including globulin (Spearman's rho = 0.32, *P*=0.007), albumin (Spearman's rho = −0.686, *P*=3.744*e* − 11), total protein (Spearman's rho = −0.74, *P*=1.639*e* − 13), and triglyceride (Spearman's rho = −0.35, *P*=0.00249, Supplementary Figure 1). Systolic blood pressure was correlated with diastolic blood pressure and age (Spearman correlations, rho = 0.5567, 0.298, and *P*=4.629*e* − 07, 0.0114, respectively). We did not find correlation between systolic blood pressure and BMI in this cohort.

### 3.2. Comparison of Gut Microbiomes across Locations

**Figure 2:**
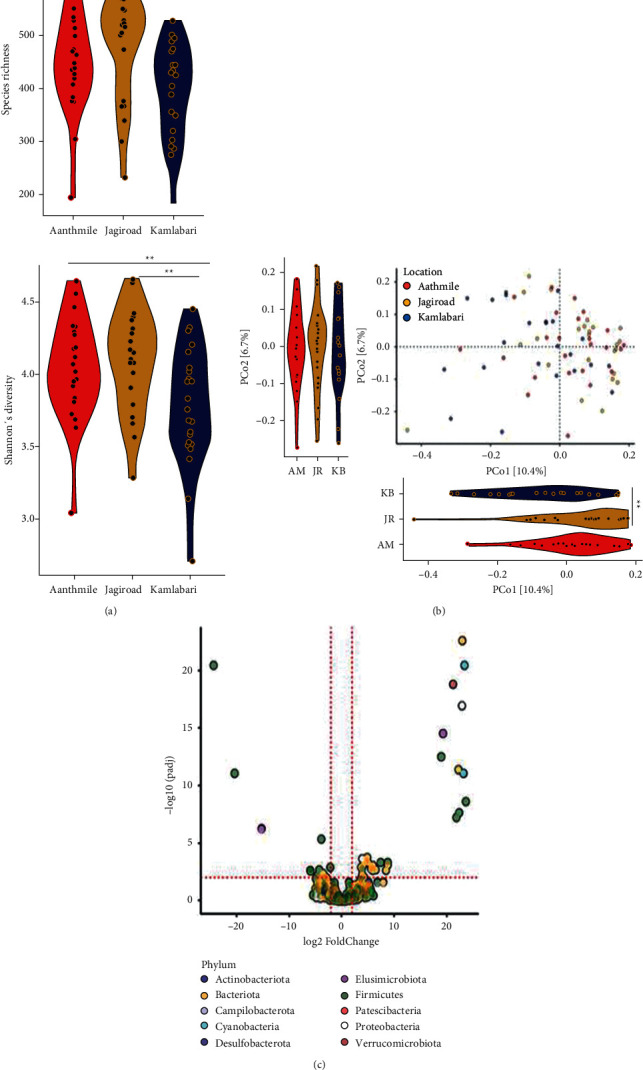
Gut microbial differences between populations. (a) Species richness and Shannon's diversity varied slightly between the three populations (*P*=0.007453 and 0.017, respectively, Kruskal–Wallis test). Species richness was significantly different between Kamalabari (KB) and Jagiroad. Shannon's differed significantly in Kamalabari population relative to the other two populations. (b) Visualization of PCoA using Bray–Curtis distance revealed that Kamalabari was significantly different from both the Nepali populations. Aanthmile (AM) and Jagiroad (JR) populations did not differ significantly. (c) A total of 44 amplicon sequence variant (ASV) level differences between the populations (compared to Kamalabari) were obtained using differential expression analysis for sequence count data version 2 (DESeq2) (^*∗∗*^<0.05).

To evaluate the effect of subtle dietary and lifestyle differences between the three populations on their GM, we compared their gut microbiome diversity and composition (Figure 2). Rarefaction curves ranging from sequencing depth of 1,000–30,000 revealed that the sequencing depth was sufficient to evaluate alpha diversity in these individuals (Supplementary Figure 2). We compared alpha diversity between populations using two standard measures, species richness and Shannon diversity index at the rarefaction depth of 30,000. Slight variations in species richness and Shannon's diversity were observed across the three locations (*P*=0.0074 and 0.017, respectively, *Kruskal–Wallis test*, Figure 2(a)). However, pairwise comparisons revealed that the species richness was significantly lower in participants from Kamalabari relative to Jagiroad only. On the other hand, Shannon's diversity index was significantly lower in participants from Kamalabari relative to those from Jagiroad as well as Aanthmile (FDR adjusted *P* < 0.05; *Dunn's post hoc test*).

Gut microbiome composition assessed using a principal coordinate analysis (PCoA) at the ASV level using Bray–Curtis also showed variations in gut microbiome composition between the three locations (*P*=1*e* − 04, PERMANOVA). Visualization of the principal coordinates (PCos) revealed subtle shifts in GM composition in Kamalabari participants. However, no difference was detectable between individuals from Aanthmile and Jagiroad (Figure 2(b)). Similar results were obtained using weighted and unweighted UniFRAC distances (Supplementary Figure 3). We also observed differences in the gut microbiomes between the individuals from the three locations at the genus level (Figure 2(c)).

Despite the statistical significance, the magnitude of gut microbiome differences between these populations was small. For instance, the effect size of location on alpha diversity measured using species richness and Shannon's diversity index was 11.9% and 12.4%, respectively, and it explained only ∼5% of variance in gut microbial composition. To further demonstrate this point, a random forest classifier was used to assess whether individuals can be classified into their respective populations based on their GM (ASVs). The overall accuracy of the classifier was low (46%), and classification error rates were 37.5%, 58.8%, and 41.2% for Kamalabari, Jagiroad, and Aanthmile, respectively (Supplementary Table 1). These results collectively indicated that there are detectable differences in the gut microbiome between these locations, but such differences are minute.

### 3.3. Gut Microbiome Is Associated with Systolic Blood Pressure

**Figure 3:**
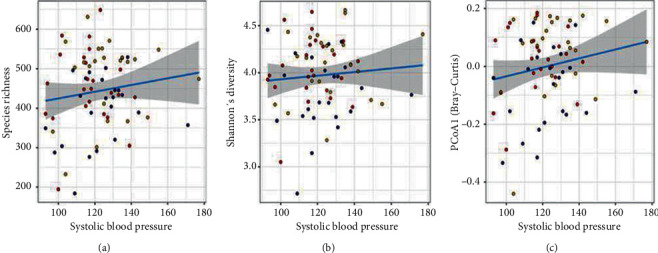
Gut microbial association with systolic blood pressure. A multivariate generalized linear mixed model (GLMM) was constructed to identify the association of gut microbe composition (random effect) with 15 host factors (fixed effect). Strong association was observed between systolic blood pressure and (a) species richness, (b) Shannon's diversity, and (c) PCo1 using Bray–Curtis distance. The line is fitted linear model.

The aforementioned analyses revealed that geographical location explained only a small fraction of the GM variation in this cohort. Therefore, we sought to identify additional factors that may be associated with gut microbiome. Few factors including consumption of rice, pulses, and vegetables were similar across the three locations, and alcohol consumption was rare. A total of 7 variables that were either invariable or redundant were removed from the dataset, and we assessed association between three measures of GM and 15 remaining host factors. These factors included location, age, sex, milk drinking pattern, gastric condition, medicinal use, systolic and diastolic blood pressure, hypertension (a categorical variable defined as systolic ≥ 140 and diastolic ≥ 90), BMI, as well as levels of albumin, globulin, triglycerides, cholesterol, and glucose in the blood. Three multivariate generalized linear mixed models were constructed for three GM measures—species richness, Shannon's diversity index, and PCo1 calculated using Bray–Curtis—where each of these 15 variables was considered to have fixed effects and random effect was assigned to each individual. In these multivariate analyses, the location was no longer significantly associated with alpha diversity (*P* > 0.05, GLMM, for species richness and Shannon's diversity both). Of the 15 factors, systolic blood pressure was the only variable that was significantly associated with both measures of alpha diversity, species richness (*P*=0.02, GLMM, Figure 3(a)) as well as Shannon's diversity (*P*=0.04, GLMM, Figure 3(b)). However, PCo1 showed significant difference between Kamalabari and Jagiroad populations (*P*=0.04, GLMM), but no differences were observed between Kamalabari and Aanthmile or Jagiroad and Aanthmile (*P* > 0.05, GLMM). After accounting for GM variation explained by location, systolic blood pressure was the only other variable associated with PCo1 (*P*=0.01, GLMM, Figure 3(c)). These analyses repeated for PCo2 revealed associations between GM and blood triglyceride levels and medicinal use (*P* < 0.05, GLMM, Supplementary Figure 4).

### 3.4. Bacterial Taxa Predictive of Blood Pressure

**Figure 4:**
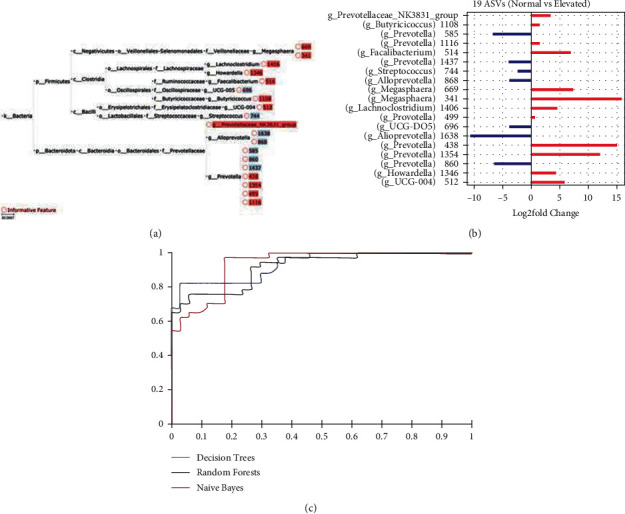
Gut bacteria associated with blood pressure. (a) Identification of 19 bacterial taxa associated with systolic blood pressure using HFE. ASVs were used as features. Systolic blood pressure was categorized into normal (<120 mmHg) and elevated (>120 mmHg), and the binary categories were used to identify bacterial features. (b) Differential relative abundance of the 19 informative ASVs between the two blood pressure groups. Red bars indicate ASVs with higher relative abundance in individuals with high systolic blood pressure, and blue bars indicate ASVs with higher relative abundance in individual with normal blood pressure. (c) Area under curve (AUC) for decision tree, random forest, and naive Bayes.

Of the 71 individuals, 37 and 34 had normal and elevated blood pressure, respectively. To identify the gut bacteria (ASVs) that distinguish individuals with normal blood pressure (systolic <120 mmHg) from those elevated (high) blood pressure (systolic ≥ 120 mmHg), three machine learning (ML)-based classifiers were tested. Hierarchical feature engineering (HFE) [34] was implemented to exploit the intrinsic hierarchical structure of the taxonomical feature of the 1935 ASVs, which resulted in 19 informative ASVs (Figure 4(a)). Twelve of these 19 ASVs had higher relative abundance in individuals with elevated blood pressure. These bacteria associated with high blood pressure included 4 ASVs from genus *Prevotella*, 2 ASVs from *Megasphaera*, and 1 ASV from *Butyricicoccus*, *Prevotellaceae*, *Faecalibacterium*, *Lachnoclostridium*, *Howardella*, and g-UCG04 (Figure 4(b)). The 7 ASVs with higher relative abundance in individual with normal blood pressure included 3 from genus *Prevotella*, 2 from *Alloprevotella*, and 1 from *Streptococcus* and g-UCG-05. Next, we tested whether these 19 ASVs could be used to accurately predict individuals with elevated blood pressure using three machine learning methods, namely, decision trees, random forests, and naive Bayes (Figure 4(c)). Naïve Bayes algorithm generated the best classifier that outperformed the two other algorithms in terms of precision with 88%, recall with 87.3%, F1-score with 87.2%, and area under the curve (AUC) with 92.8%. As an alternative approach, DESeq2 analysis performed using the same dataset revealed a total of 9 differential ASVs between the two groups (Supplementary Figure 5), 2 of which were also identified by HFE.

### 3.5. Blood Pressure vs. Firmicutes-to-Bacteroidetes Ratio

**Figure 5:**
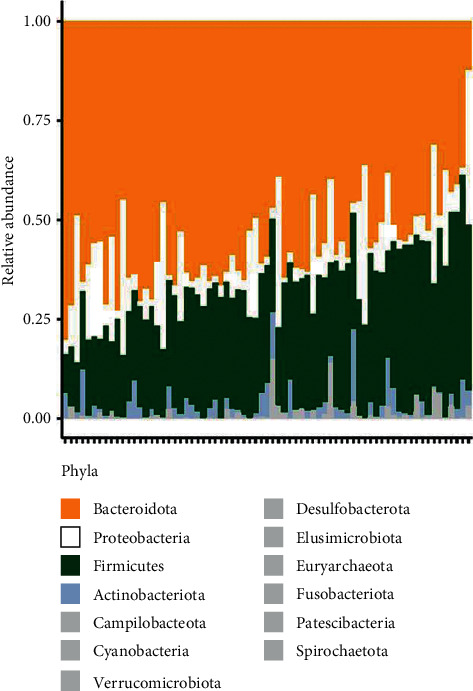
Gut microbiome composition among individuals. Relative abundances of each phylum across every individual represented by stacked bars. Four most significant phyla are shown in individual colors. All other phyla are grouped as “Other,” and their combined relative abundances are represented in grey. The relative abundance of Firmicutes ranged from ∼2% to 40%, while abundance of Bacteroidetes ranged from ∼75% to ∼25%.

Previous studies have suggested that the relative abundance of Firmicutes-to-Bacteroidetes ratio (F/B) is associated with different measures of health and diseases, including hypertension [15]. Firmicutes and Bacteroidetes were also the two most dominant phyla in this cohort (Figure 5). The relative abundance of Firmicutes ranged from ∼2% to 40%, while abundance of Bacteroidetes ranged from ∼75% to ∼25%. We found that F/B was strongly negatively correlated with Shannon's diversity index (Spearman's rho = −0.32, *P*=0.008). However, a multivariate model including the 15 variables showed that F/B ratio was not significantly associated with systolic blood pressure (*P*=0.4797, GLMM).

## 4. Discussion

Human gut microbiome mirrors lifestyle [38, 39] and can have profound influence on human health [40]. Although several studies have investigated the link between the gut microbiome and human diseases, those studies have primarily focused on Western or industrialized populations [1]. Given the gut microbiota of traditional populations differ significantly, whether the findings in industrialized populations can be extended to traditional populations remains unknown. Here, we compare gut microbiomes from 71 individuals belonging to two different agropastoral ethnicities that cohabit three small geographical areas in Assam, Northeastern India. Our survey results showed that these populations have subtle variation in lifestyle and dietary habits. Although the primary sources of carbohydrates and proteins as well as fiber intake via vegetable consumption are similar between these populations, there are few subtle variables linked to cultural practices that are strongly linked to the two ethnicities. For example, all the participants consumed *doi* (yogurt). While *doi* is prepared by natural fermentation of raw milk in the native Assamese community in Kamalabari, it is prepared by propagation of cultures in boiled milk in the Nepali-speaking villages in Aanthmile and Jagiroad. Similarly, the consumption of milk was lower in Kamalabari and their primary source of animal protein was fish while meat was the primary source of animal protein in the other two villages. We also observed differences in a few blood biomarkers between the native Assamese and participants from the other two locations. Serum albumin and total protein levels were lower in the Nepali speakers relative to the Assamese population, but serum globulin level was lower in the Assamese. These findings are consistent with a previous study [36]. Higher serum albumin and total protein are biomarkers for poor nutritional status [41]. On the other hand, higher globulin may indicate poor hygienic conditions [36]. These findings indicate that lifestyle guided by the Neo-Vaishnavite principles, which emphasizes pescatarian diet, good hygiene, and meditation, is reflected in the Satra's blood biochemistry.

Previous studies comparing populations with distinct lifestyles have identified large differences in gut microbiomes [2, 42], but whether subtle lifestyle variations manifest in detectable gut microbiome differences in geographically cohabiting populations remained unexplored. In this study, we found that the subtle dietary differences between the participants reflected in small but detectable variations in their gut microbiota. The gut microbiome diversity and composition were slightly but consistently different between the native Assamese and the two Indo-European communities in Aanthmile and Jagiroad. But neither diversity nor composition differed significantly between the participants of Aanthmile and Jagiroad, indicating that the gut microbiota variations mirrored the lifestyle differences between these communities.

In addition to lifestyle, we found a robust link between gut bacteria and blood pressure in these participants as both measures of alpha diversity and beta diversity measured using the primary axis in a principal coordinate analysis (PCo1) were associated with systolic blood pressure. Furthermore, integration of a machine learning approach identified 19 ASVs that were predictive of elevated blood pressure in this cohort. Importantly, many of these 19 ASVs have been associated with hypertension in previous studies. Also, the direction of change in relative abundance of these ASVs in individuals with high blood pressure in this study is consistent with hypertension patients. For example, four of the ASVs elevated in individuals with high blood pressure belonged to the genus *Prevotella*, which has also been shown to be overrepresented in individuals with prehypertension and hypertension in non-Indian populations [43]. *Prevotella* may play an essential role in hypertension by triggering inflammatory response [43]. Similarly, we observed 1 ASV from genus *Lachnoclostridium* that had higher relative abundance in individuals with elevated blood pressure, which is consistent with previous findings where *Lachnoclostridium* was reported to be positively associated with systolic blood pressure [44]. Two *Megasphaera* ASVs were elevated in the high blood pressure group. *Megasphaera* is a common commensal found in the Indian gut [45], and it has previously been reported to be significantly abundant in hypertensive individuals [46]. Finally, initial studies suggested increase in F/B ratio and decrease in microbial diversity as signatures of hypertension [18, 43], which was contradicted by a recent study with large cohort of Western individuals [1]. Consistent with the latter study, F/B ratio was not significantly associated with systolic blood pressure in our study as well. Although our results recapitulated many observations corroborating previous links between gut microbiome and hypertension, there was one contradiction. A *Faecalibacterium* ASV was higher in individuals with elevated systolic blood pressure. *Faecalibacterium* is an important carbohydrate fermenting bacteria involved in butyrate production via starch fermentation [6], and lower relative abundance of this genus has been associated with hypertension in previous findings [43, 47].

Our study has revealed that systolic blood pressure is associated with gut microbiome in an Indian population. This finding is important for two reasons. First, it demonstrates that gut bacteria can be linked to chronic diseases in traditional populations. Traditional populations have been historically underrepresented in genomics studies [48, 49], and this trend is starting to extend to microbiome studies. Several large cohorts consisting of thousand participants from industrialized populations have successfully identified factors associated with gut microbiome and health [1, 41, 50]. Yet, very few studies have attempted to investigate the link between microbiome and health in traditional populations. Given both the lifestyle and gut bacteria of traditional populations differ from industrialized populations, lack of microbiome-disease connections in traditional populations has the potential to augment the already existing health disparities in genomic medicine. Second, the findings from this study are highly relevant to Indian populations. Hypertension is highly prevalent in India, and in Assam, it is the primary risk factor for cardiovascular diseases. High blood pressure currently affects 1.3 billion individuals in India—which is 45% of the entire Indian adult population [21]—increasing their risk of developing cardiovascular diseases. The findings from this study pave way for future investigations involving larger cohorts designed to develop personalized, preventative, and targeted interventions harnessing the gut microbiome to tackle the burden of cardiovascular diseases.

## 5. Conclusion

Microbiome has become increasingly important biomarker in framing novel strategies to address etiology of several diseases. Lack of microbiome-disease connections in traditional populations has the potential to augment the already existing health disparities in genomic medicine. In India, Assam is home to several ethnic tribes who still practice traditional subsistence while hypertension is the leading cause of cardiovascular diseases in this region. The result presented herein is the first report to address the association of systolic blood pressure with gut microbiome of two different agropastoralist ethnic communities. Compositional profiling revealed that gut microbiome of normal and elevated hypertensive individuals has significantly different amplicon sequence variants. These findings confer opportunities to design personalized, preventative, and targeted interventions harnessing the gut microbiome to tackle the burden of cardiovascular diseases.

## Figures and Tables

**Table 1:** Systolic and diastolic blood pressure in male vs. female.

Attributes	Overall	Male	Female
Total participants	71	51	20
Systolic	122.32 ± 16.47	122.3 ± 17.69	122.3 ± 13.26
Diastolic	71.39 ± 11.86	70.52 ± 12.44	73.6 ± 10.18
Sex	100	71.80%	28.20%
Age	35.9 ± 11.4	34.9 ± 10.4	38.4 ± 12.4
BMI	19 ± 6.1	19.00 ± 7.93	24.56 ± 6.05

## Data Availability

The dataset analyzed during the current study are available in MG-RAST repository under the project id mgp85002 and mgp85087 (Supplementary Table 2).

## Supplementary Materials

Supplementary Materials Supplementary Figure 1: correlations between variables, including diet and blood parameters in Assamese populations. Supplementary Figure 2: alpha diversity across the locations. Supplementary Figure 3: visualization of gut microbial compositions across the locations using principal coordinate analysis (PCoA). Supplementary Figure 4: principal coordinate analysis (PCoA 2) using Bray–Curtis distance. Supplementary Figure 5: gut bacteria associated with blood pressure. Supplementary Table 1 presents random forest classification of 16S data.
