# Supplementary material for: Human Gut Microbes Associated with Systolic Blood Pressure
Source: Int J Hypertens. 2022 Feb 3;2022:2923941. doi: 10.1155/2022/2923941 (PMC8831042; doi:10.1155/2022/2923941)
Supplement: Supplementary Materials — Supplementary Figure 1: correlations between variables, including diet and blood parameters in Assamese populations. Supplementary Figure 2: alpha diversity across the locations. Supplementary Figure 3: visualization of gut microbial compositions across the locations using principal coordinate analysis (PCoA). Supplementary Figure 4: principal coordinate analysis (PCoA 2) using Bray–Curtis distance. Supplementary Figure 5: gut bacteria associated with blood pressure. Supplementary Table 1 presents random forest classification of 16S data. [file 2923941.f1.docx]

**Supplementary figures**

**Supplementary Figure. 1** **Correlations between variables, including diet and blood parameters in Assamese populations**. Systolic BP and BMI were not strongly correlated in this dataset (Spearman’s rho = 0.17, *P*=0.16).


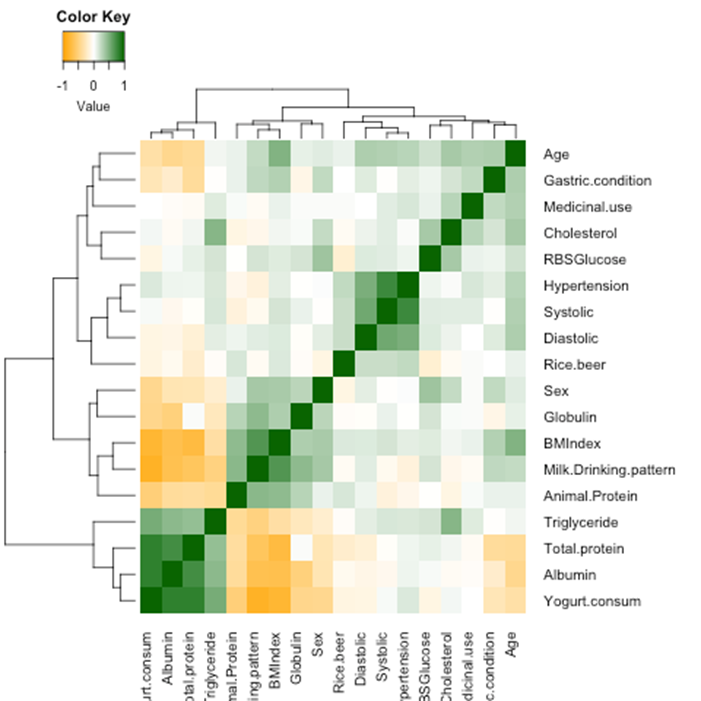


**Supplementary Figure. 2** **Alpha diversity across the Locations**. Rarefaction curves depicting two commonly used measures including species richness (Top) and shannon diversity index (bottom) were calculated by subsampling 2500-30000 reads per sample.


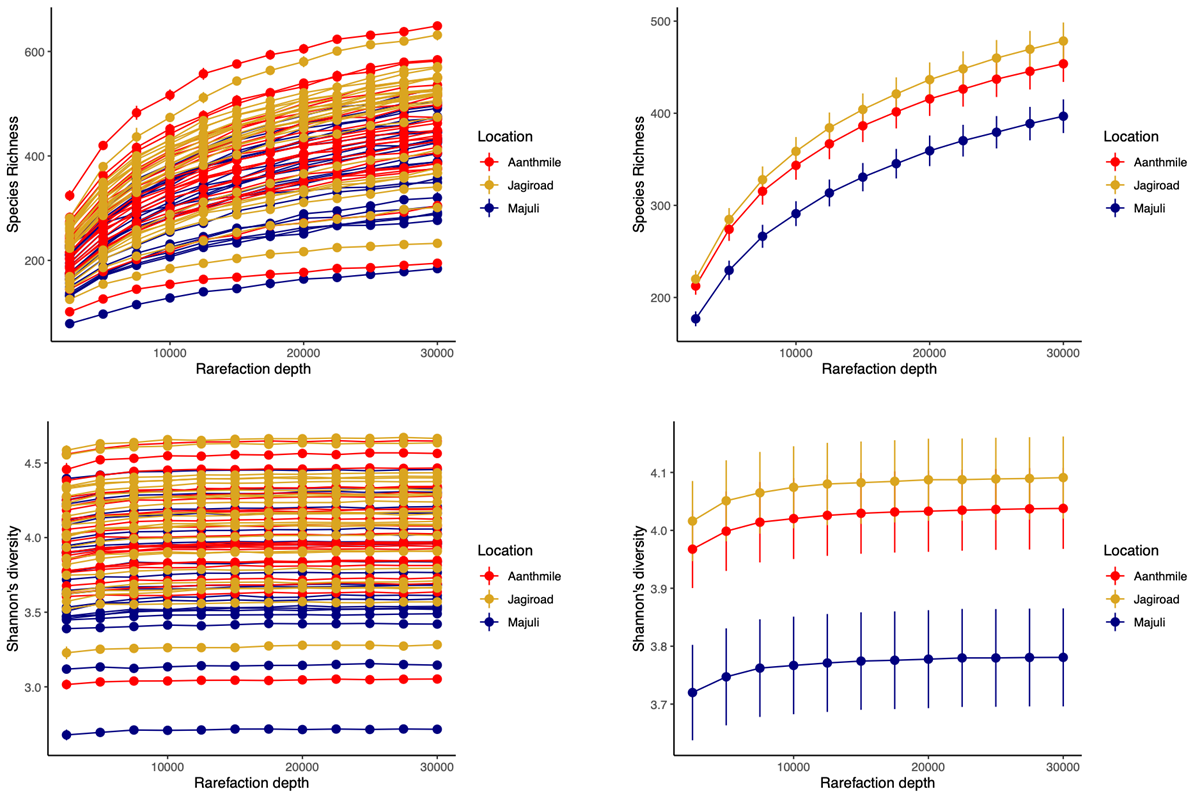


**Supplementary Figure. 3** **Visualisation of gut microbial compositions across the locations using Principal coordinate Analysis (PCoA)**. The PCoA of the (a) unweighted and (b) weighted Unifrac distance.


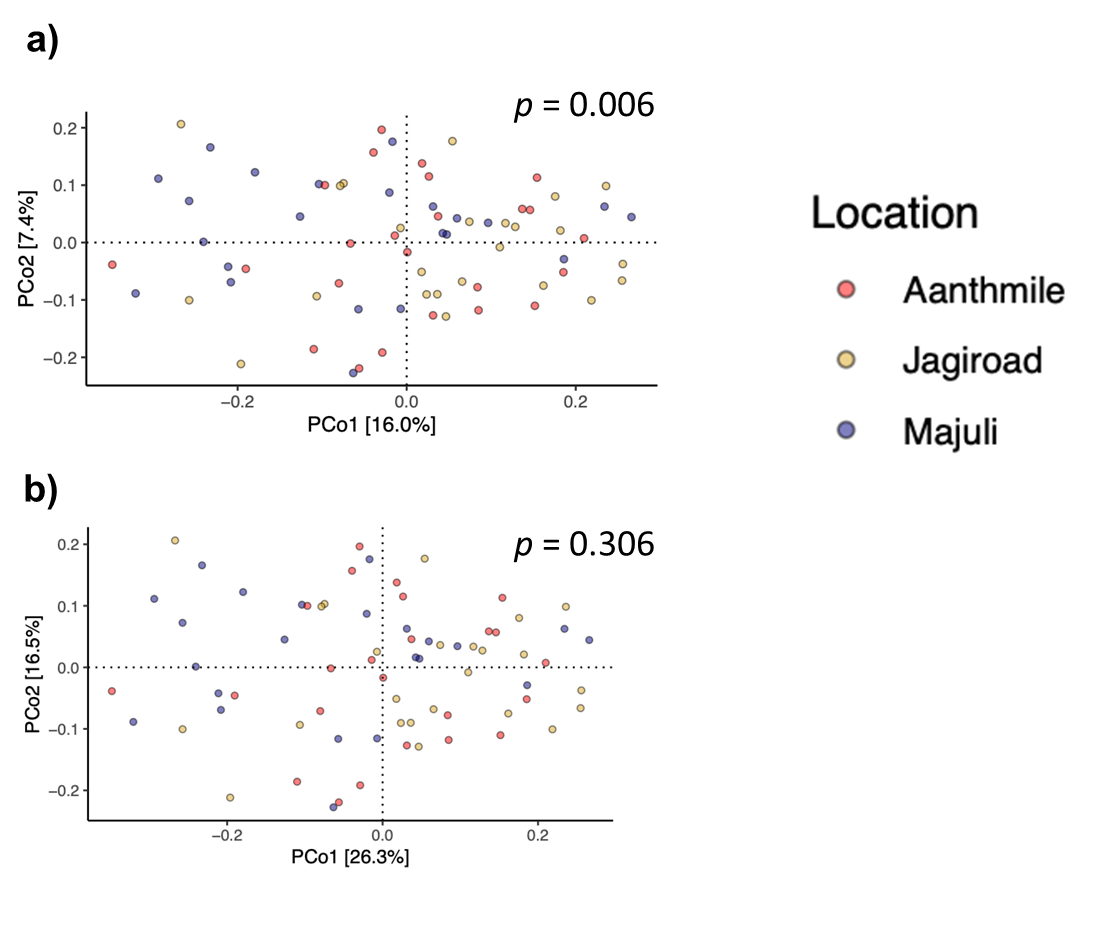


**Supplementary Figure. 4** Principal Coordinate analysis (PCoA 2) using bray curtis distance were associated with (A) Medicinal use and (B) Triglycerides.


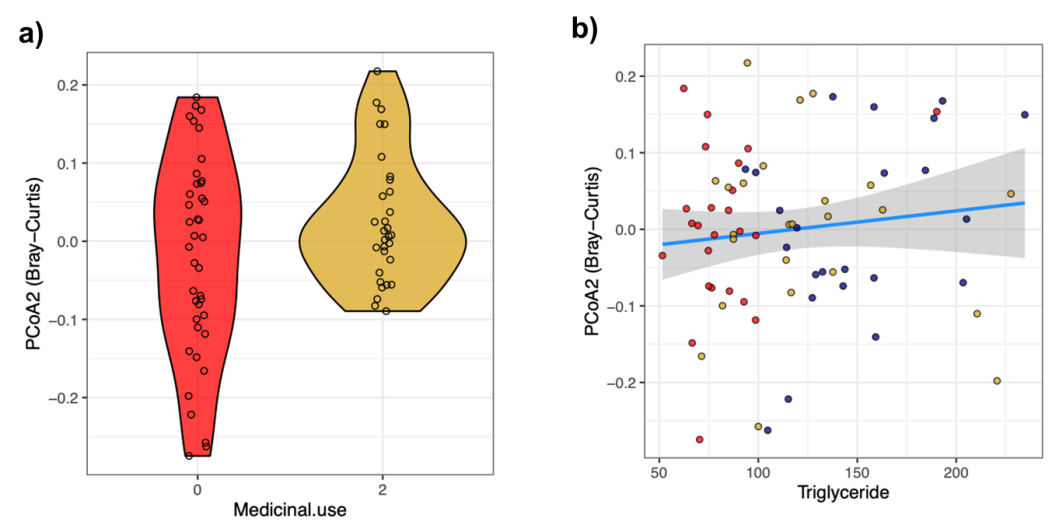


**Supplementary Figure. 5** **Gut bacteria associated with blood pressure.** To test whether the features identified in the machine learning approach could also be identified as significant using an alternative method, we performed differential analysis using DESeq2. We identified 9 significant taxa (*p*adj<0.05 and absolute l2FC >1.5).


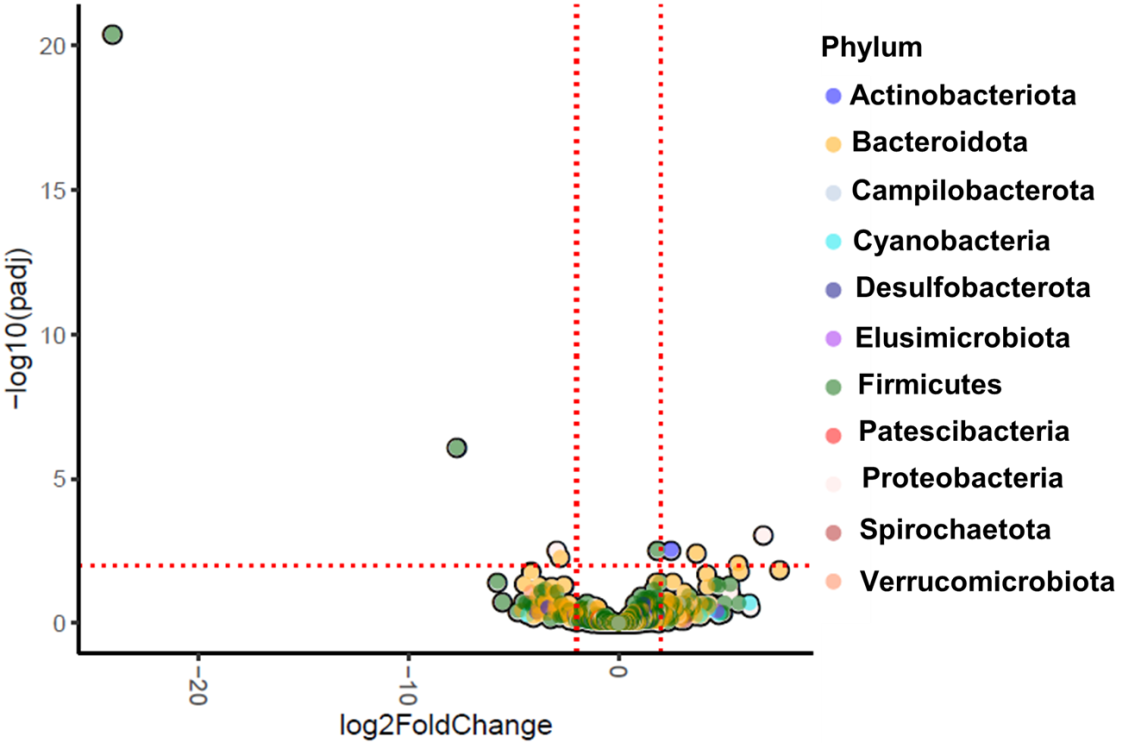


**Supplementary tables**:

**Supplementary Table 1**

Random forest classification of 16 S data

|  | Aanthmile | Jagiroad | Majuli | Class.error |
| --- | --- | --- | --- | --- |
| Aanthmile | 10 | 5 | 2 | 0.411 |
| Jagiroad | 7 | 7 | 3 | 0.588 |
| Majuli | 2 | 4 | 10 | 0.375 |

Out of bag (OOB) estimate of error rate: 46%

Supplementary Table 2

**Sample code and MG-RAST ID of the sequences submitted in MG-RAST database**

| **Sl. No.** | **Location** | **Community** | **Sample ID** | **MGRAST ID** |
| --- | --- | --- | --- | --- |
| 1 | Aanthmile | Nepali | 342 | a3e66a7c116d676d343739343330332e33 |
| 2 | Aanthmile | Nepali | 343 | 6ef2bb648f6d676d343739343239352e33 |
| 3 | Aanthmile | Nepali | 344 | f8cdc06a576d676d343739343239302e33 |
| 4 | Aanthmile | Nepali | 345 | d1a8214de16d676d343739343237302e33 |
| 5 | Aanthmile | Nepali | 346 | e358693e206d676d343739343238372e33 |
| 6 | Aanthmile | Nepali | 347 | 59b6bb32076d676d343739343331302e33 |
| 7 | Aanthmile | Nepali | 348 | 1c82f68c276d676d343739343237352e33 |
| 8 | Aanthmile | Nepali | 349 | 4d4ee1ff9f6d676d343739343237332e33 |
| 9 | Aanthmile | Nepali | 350 | 5c4a6223476d676d343739343330312e33 |
| 10 | Aanthmile | Nepali | 351 | d75d177cee6d676d343739343237382e33 |
| 11 | Aanthmile | Nepali | 352 | 03b59ce6336d676d343739343238392e33 |
| 12 | Aanthmile | Nepali | 353 | f4be4094386d676d343739343236332e33 |
| 13 | Aanthmile | Nepali | 354 | d5996f5ef96d676d343739343330372e33 |
| 14 | Aanthmile | Nepali | 355 | 866acc7ebe6d676d343739343237362e33 |
| 15 | Aanthmile | Nepali | 356 | 8f026447fc6d676d343739343237312e33 |
| 16 | Aanthmile | Nepali | 357 | 971524ea916d676d343739343330302e33 |
| 17 | Aanthmile | Nepali | 358 | e82a26da2c6d676d343739343236392e33 |
| 18 | Aanthmile | Nepali | 359 | aef6822d3a6d676d343739343330362e33 |
| 19 | Aanthmile | Nepali | 360 | 9932bff7ba6d676d343739343236372e33 |
| 20 | Aanthmile | Nepali | 361 | 280ce588906d676d343739343239382e33 |
| 21 | Aanthmile | Nepali | 363 | 7631bbd23d6d676d343739343239372e33 |
| 22 | Aanthmile | Nepali | 364 | 2f673a2c0c6d676d343739343239362e33 |
| 23 | Aanthmile | Nepali | 365 | b0791de30d6d676d343739343239312e33 |
| 24 | Aanthmile | Nepali | 366 | 0f1cec5d576d676d343739343238362e33 |
| 25 | Jagiroad | Nepali | 367 | bba47d9f786d676d343739343236382e33 |
| 26 | Jagiroad | Nepali | 368 | 5a6ea9dfdb6d676d343739343236352e33 |
| 27 | Jagiroad | Nepali | 369 | 04627819076d676d343739343238352e33 |
| 28 | Jagiroad | Nepali | 370 | 85a02149dd6d676d343739343330382e33 |
| 29 | Jagiroad | Nepali | 371 | e2f3f51c516d676d343739343330352e33 |
| 30 | Jagiroad | Nepali | 372 | 9fd0c04a2d6d676d343739343238302e33 |
| 31 | Jagiroad | Nepali | 373 | c80543b9106d676d343739343239322e33 |
| 32 | Jagiroad | Nepali | 374 | 1fb3be0ddd6d676d343739343330342e33 |
| 33 | Jagiroad | Nepali | 375 | 717f7b6b926d676d343739343237322e33 |
| 34 | Jagiroad | Nepali | 376 | b635a910016d676d343739343239332e33 |
| 35 | Jagiroad | Nepali | 377 | 54e1c712c26d676d343739343238312e33 |
| 36 | Jagiroad | Nepali | 378 | d2b5d0ed7d6d676d343739343238342e33 |
| 37 | Jagiroad | Nepali | 379 | c671d8abd26d676d343739343332342e33 |
| 38 | Jagiroad | Nepali | 380 | a94f5dd96e6d676d343739343236322e33 |
| 39 | Jagiroad | Nepali | 381 | 9d06c0641e6d676d343739343238322e33 |
| **Sl. No.** | **Location** | **Community** | **Sample ID** | **MGRAST ID** |
| 40 | Jagiroad | Nepali | 382 | f82e4c76c86d676d343739343239392e33 |
| 41 | Jagiroad | Nepali | 383 | 4ab3845d476d676d343739343238382e33 |
| 42 | Jagiroad | Nepali | 384 | f3eb3d05dc6d676d343739343237392e33 |
| 43 | Jagiroad | Nepali | 385 | 8cf35dc4ab6d676d343739343236362e33 |
| 44 | Jagiroad | Nepali | 386 | 96304b5d8e6d676d343739343237342e33 |
| 45 | Jagiroad | Nepali | 387 | f9e8607e196d676d343739343330392e33 |
| 46 | Jagiroad | Nepali | 388 | 7a6535fe186d676d343739343330322e33 |
| 47 | Jagiroad | Nepali | 389 | d405d52ea36d676d343739343238332e33 |
| 48 | Jagiroad | Nepali | 390 | e4d5aeb9fc6d676d343739343239342e33 |
| 49 | Kamlabari | Satra | 277 | mgs656469 |
| 50 | Kamlabari | Satra | 278 | mgs656472 |
| 51 | Kamlabari | Satra | 279 | mgs656475 |
| 52 | Kamlabari | Satra | 280 | mgs656478 |
| 53 | Kamlabari | Satra | 281 | mgs656481 |
| 54 | Kamlabari | Satra | 282 | mgs656484 |
| 55 | Kamlabari | Satra | 283 | mgs656487 |
| 56 | Kamlabari | Satra | 284 | mgs656490 |
| 57 | Kamlabari | Satra | 285 | mgs656493 |
| 58 | Kamlabari | Satra | 286 | mgs656496 |
| 59 | Kamlabari | Satra | 287 | mgs656499 |
| 60 | Kamlabari | Satra | 288 | mgs656502 |
| 61 | Kamlabari | Satra | 289 | mgs656505 |
| 62 | Kamlabari | Satra | 290 | mgs656508 |
| 63 | Kamlabari | Satra | 291 | mgs656511 |
| 64 | Kamlabari | Satra | 292 | mgs656514 |
| 65 | Kamlabari | Satra | 293 | mgs656517 |
| 66 | Kamlabari | Satra | 294 | mgs656520 |
| 67 | Kamlabari | Satra | 295 | mgs656523 |
| 68 | Kamlabari | Satra | 296 | mgs656526 |
| 69 | Kamlabari | Satra | 297 | mgs656529 |
| 70 | Kamlabari | Satra | 298 | mgs656532 |
| 71 | Kamlabari | Satra | 300 | mgs656538 |
